# Patients’ Perspective of the Design of Provider-Patients Electronic Communication Services 

**DOI:** 10.3390/ijerph110606231

**Published:** 2014-06-12

**Authors:** Petr Silhavy, Radek Silhavy, Zdenka Prokopova

**Affiliations:** Department of Computer and Communication Systems, Faculty of Applied Informatics, Tomas Bata University in Zlin, nam. T. G. Masaryka 5555, Zlin, 760 01, Czech Republic; E-Mails: psilhavy@fai.utb.cz (P.S.); prokopova@fai.utb.cz (Z.P.)

**Keywords:** electronic communication, portal design, web-based healthcare

## Abstract

Information Delivery is one the most important tasks in healthcare practice. This article discusses patient’s tasks and perspectives, which are then used to design a new Effective Electronic Methodology. The system design methods applicable to electronic communication in the healthcare sector are also described. The architecture and the methodology for the healthcare service portal are set out in the proposed system design.

## 1. Introduction

Web-based portals are assuming an ever-more important role in the medical application development field. Their importance and usability are increasing rapidly as the web is becoming an appropriate eco-system for medical information portals. These systems use the client-server approach, because of their support for high levels of productivity and scalability. 

The web is only one of many possible ways of delivering information. For medical information delivery purposes, the touch-screen kiosk is a very popular tool. Discussion between medical professionals and academic researchers about the advantages and disadvantages of this approach is ongoing, mainly about if such a system can be trusted. Rauer’s [1] investigations indicated that trust in electronic records varies, with patients having a greater tendency to trust them than professionals. This discussion is closely connected to the development of information technology and patients’ needs to receive more and more information. 

A web-based portal is based on web technologies. The Internet network is used for communications between a patient and the application. The most important advantage of a web-based portal is the possibility of connecting with personal computers at home or in the office. Web portals provide the possibility of integrating several differing information sources.

In [2] the authors presented a study based on 1172 interactions between stakeholders concerning 100 patients who were randomly selected. The results show that usage of the portal is only random. They did not investigate why usage of the portal was low. 

Bashshur [3] describes telemedicine in healthcare. These results should be used for Internet service in general. Kohane *et al.* [4] describe methods for building an electronic health records system. These findings are useful for an understanding of web technologies in healthcare, but they only describe one part of a web-based portal. Therefore, their research is expanded in this paper and further discussion is engaged. 

Provider’s needs may vary in relatively huge interval terms. There are systems for clinical care, information exchange, and service request functions, whose integration into existing patient-care and communication workflows requires careful planning. Later in the paper, the authors address patient requirements. Modern Internet users are looking for user-friendly and information intensive systems, which they are used to in other sectors. Providers are looking for modern and secure systems, which can help cost-management and can help to build a cost-effective healthcare system at the national level. According to study [5], reducing face-to-face communication is one possible approach, which increases the efficacy of the healthcare’s systems, for example, by allowing those patients who need complex monitoring or regularly examination to ask for a remote communication solution. In [6,7,8], the authors present the fact that patients can omit some important information or they can fail to remember what the physician told them. 

The aim of this paper is to present a methodology showing how such a communication system can be designed. The authors believe that, by using the results of this investigation, it is possible to improve web-based frameworks for portals in the healthcare sectors. The organization of this contribution is as follows: Section 2 describes the basic experimental prerequisites. Section 3 describes the results and provides a discussion of those results. Finally, Section 4 gives the conclusions.

## 2. Methodology Prerequisites

The architecture that is proposed in this article is built according to user needs, which were ascertained from a user survey. Healthcare providers are trying to both improve communication ability and reduce loading capacity. They have therefore begun by implementing provider-patients web-based portals [7,8,9,10,11]. 

The communication methods which are typically involved include: e-mail, appointment scheduling, and medication refill requests. In addition, the provider’s information system supports transfers of clinical data. In the healthcare sector, the FIFO-based system is the most popular approach, *i.e.*, using the chronology of the attendance of waiting patients. This causes long queues in the waiting rooms. There is no chance to control either the time of the patients, nor that of the providers. Although there is the possibility of telephone orders, but very often, patients miss their examination appointments. 

The electronic prescription is a specific form of provider-patient communication. This form is based on an electronic request for a medicament prescription. Nowadays, typically only a physical visit to the health-care provider’s office leads to the prescription of medicaments. This is highly time-consuming. The second aspect is, of course, the control of interference with or misuse of the medicaments prescribed. 

The possibility of electronic consultation is the third load-bearing pillar in using web portals and technology. Web portals—as a usual web-based application, allow the transfer of multimedia information—e.g., photographs, or text information only. In the written form, both patient and provider should have the opportunity to better rethink their questions and answers. 

The main advantage of solving the above-mentioned topics by exploiting web portals’ potential is also the possibility of asynchronous communication. Asynchronous communication adds a time aspect to the conversations. The main purpose of this contribution is to introduce the research goals and patient requests for such an application. Some studies [5,6,7,8,9,10] present the results of the patient’s requests:

1. Electronic consultations
2. Shortened waiting time for an examination
3. Simplification of prescriptions
4. Reminder services and invitations for preventive examinations

It is critically important to solve the first requirement precisely. Patients can report severe symptoms. This is taken as a key component of the system. Electronic communication use can be restricted by process regulations, which will declare that the system cannot be used in an emergency. 

The second requirement should be achieved by a built-in scheduler, which allows one to choose the time of the examination/appointment, which is the best for both patient and provider. If adequate time for examination is reserved, the number of patients in the queue will rapidly decrease. Patients should not need to come in advance. 

The third, simplification of the prescription process, is based on electronic prescription requests. The prescription should be delivered to the selected chemist or it should be collected in the health-care provider’s office. 

The last patient request is connected to the automation of sending invitations to attend preventive examination appointments, for vaccinations or as a reminder service. This request, probably, is based on patients’ inability or the impossibility of tracking these important tasks, but patients do feel the obligation to take part in these processes.

As can be seen in [7,11,12,13,14,15,16,17], describing the electronic system, such electronic visits can potentially improve communication, reduce frustration, and enhance relationships between patients and physicians (or the provider itself). Furthermore, patient satisfaction is increased significantly in situations when non-personal communication can help with information sharing. In the following text, the authors propose a methodology which represented an improved version of web-messaging, combined with a secure form of e-mail communication over a web portal. 

The proposed approach allows patients and providers, separated by both space, and time, to share information asynchronously in the timeslots that are usually unused for daily scheduled tasks. This is an important fact, resulting from prior study [5,7,11]. Traditional electronic communication (electronic mail) cannot be used. 

### 2.1. Methodology Design

**Figure 1:**
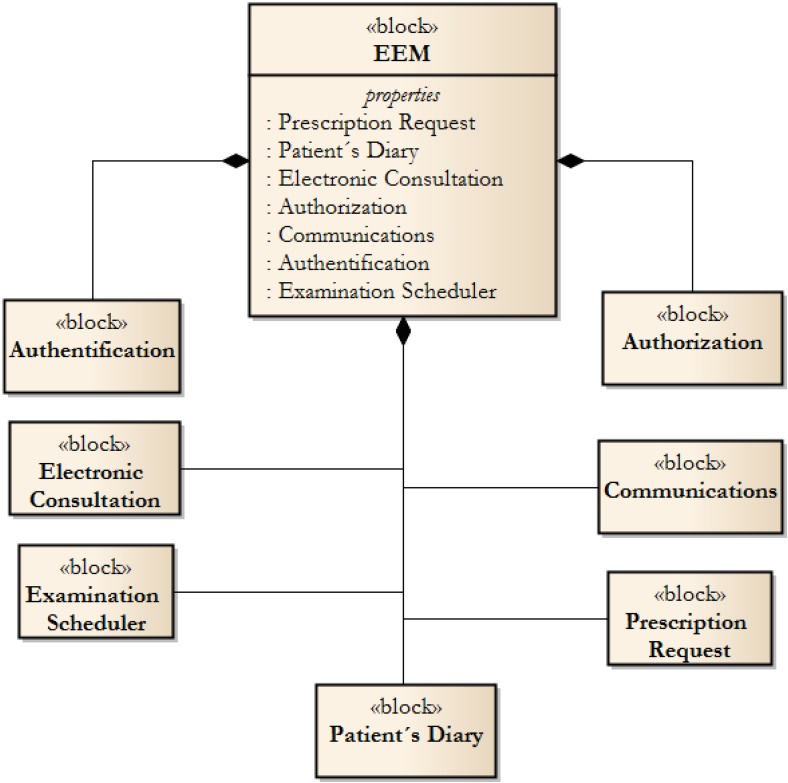
Basic blocks of the EEM.

The Effective Electronic Methodology (EEM) describes the technical aspects of services, which lead to extremely effective and fault-tolerant communication in the healthcare industry with respect to industry standards. The EEM should be understood as a guide for designing a patient-provider communication system, as a formal description of electronic services which can be implemented. The EEM is focused to be a conceptual contribution, which can be used for software design. The EEM comprises the following blocks, whose composition is illustrated in Figure 1.

The *Authentication* block is the part which describes the principle of authentication. The implementation is left open. The authors recommend using methods which originate in and from the national system standards. In the scope of the Czech Republic, two solutions are available. Firstly, the National Health Insurance Card can be used; and secondly, so-called “Data Box/Safes” can be used. Data Safes represent the state-level authentication system, which guarantee the identification of the Data Safe holder. 

The *Authorisation* block is responsible for granting rights for using the service portal functionality—blocks No. 4–8. The EEM is based on a central server-based node, which can be accessed at the general level. This means that authorized users can use its functions, upon authentication. 

The third block, *Communication*, stands for those services which are used for the generation of messages in the form of web-service messages, electronic mail messages or short text messages. 

The *Electronic Consultations* and *Patient’s Diary* are core blocks which can be used by patients. The Electronic Consultation function allows patients to submit requests, which are created *ad hoc*, or by using the Patients Diary entry function. The Patient’s Diary is used for entering information by patients or providers. Entries modification is restricted. Therefore only the user who creates the entry can change it, thus, entries which have been read cannot be modified. This improves communication consistency. 

The *Examination Scheduler* is a block which combines ordering and calendar services. As can be seen in Section 2—Methodology Prerequisites, requests for improved scheduling systems are one of the most important needs. The block is based on a scheduling calendar, which shows gaps in the health-care provider’s office hours. The methodology contains a list of attributes that can be used as a subject of examination or appointment. 

The last block, *Prescription Requests*, is used for remote requests to the health-care provider to issue new prescriptions. This service is only available for chronically-medicated patients and can (by using the Communication block) be interconnected with e-prescription services. 

### 2.2. The Primary Use Cases Model

**Figure 2:**
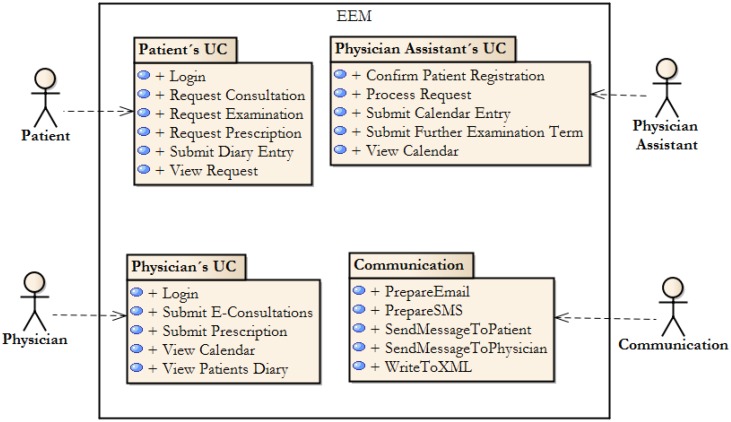
Primary use cases’ packages, which are used in EEM.

The basics blocks are described in the form of user cases. Based upon the results of their investigations, the authors have clustered the functionality of the basic blocks by system actors. In Figure 2, the Package Diagram, representing the overview of the methodology functionality can be seen. The basic functionalities, described by the methodology, can be found here. The methodology is designed as open, and therefore, it can be expanded as needed in various differing healthcare systems. 

Therefore, the Effective Electronic Methodology is based on the existence of four actors, which are triggers for the basic functionality of the system (Table 1). 

**Table 1:** List of primary actors.

Actor Name	Description
Patient	Represents the general public, can access functionality for patients
Provider	This is an actor, who represents the general provider, and can use a set of communication or data entry functions
Provider Assistant	A general actor, who stands for healthcare centres’ administrative clerks. They deal with examination planning and scheduling
Communication Gateway	A system actor, which is responsible for communication. This deals with preparing messages, which are then dispatched as web service xml, email or SMS messages

### 2.3. Web-service Based Structure

**Figure 3:**
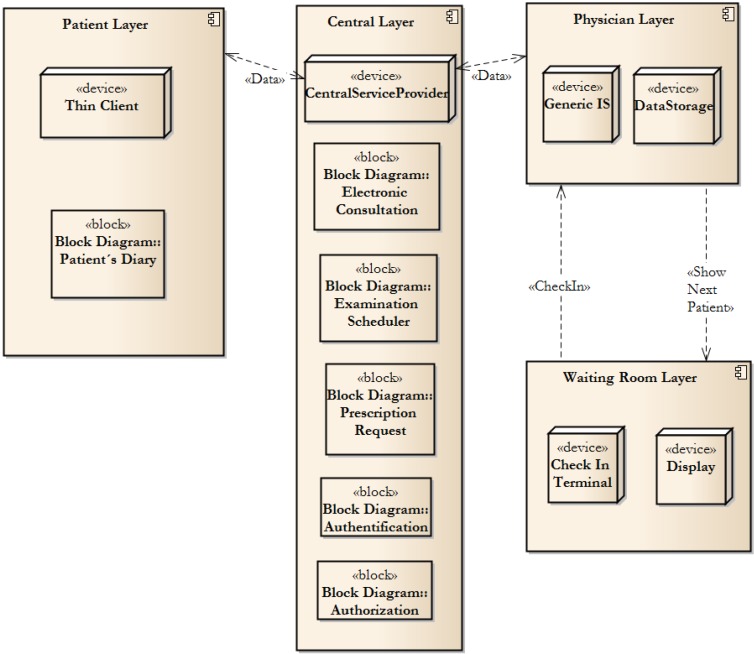
System structure.

The Effective Electronic Methodology is based on shared communication using a web service (Central Layer, see Figure 3). Healthcare providers use their own information system, to which the data exchange layer is added. A generic Internet connection is described as a data exchange layer. This leads to an extremely cost-effective solution. In Figure 3, the generic methodology structure can be seen there. The methodology philosophy is to create a Central Layer, which will act in the role of Service Provider. Only samples of basic building blocks are depicted in Figure 3. The authors propose leaving data to be stored in individual information systems, on the provider side (*i.e.*, Provider Layer). The security and accessibility of the data is better than that in a central data store. Moreover, the system is resistant to network failure.

The Central Layer contains all of the blocks shown in Figure 1. The authors propose these blocks, which are the result of an analysis of care users’ requirements. 

## 3. Results and Discussion

The Effective Electronic Methodology contributes to remove issues in healthcare service portal design, as were discussed in Section 1—Introduction. The main result can be found in the synergic effect of the architecture, which is based on web services. Therefore, the methodology is platform independent and can be used as a central electronic communication system node in the heterogonous healthcare domain.

This methodology implements the most needed parts, which are described in basic building blocks. The authentication and authorization blocks implement Rights Management and are able to grant access to specific functions that the individual healthcare centre wants to offer their patients. Furthermore, the methodology is based on parity—a 1:1 relation between the physical patient and users of the system. Therefore, it is possible to share data among healthcare centres or among independent providers. 

The blocks represent system components. Therefore, the communication block can be understood as a broker, who offers message composing services. Individuals—*i.e.*, patients, providers or healthcare centres, can configure different methods of data transfer. The web-based services are used as an inbound channel. Therefore, various information systems can be connected to a central server. We can see the similarity to a common-object-request broker architecture, which represents an independent layer.

The Electronic Consultation block brings a new standard for solving less-serious patient’s needs. The authors believe that some health-care provider tasks, mainly those related to chronically-ill patients can be performed by using remote monitoring or by electronic communication. 

Patient records in the diary support Electronic Consultation. This is the task of the Patient Record block. This is not a regular medical record, but takes the form of relatively free-form notes of personal feeling and issues which are described by the patients. 

The availability of on-line examination scheduling allowing patients’ connection to the health-care provider’s planning calendar helps to reduce waiting time. Patients are requested to check-in, when they arrive at the health-care provider’s office. In the methodology, the authors propose using medical insurance ID cards, which are issued to everyone in most countries. However, due to the open component architecture, it is also possible to implement several other authentication methods. 

In their methodology, the authors suppose an integration of several e-prescription services. Patients should be able to ask for a prescription—as described in the Prescription Request block, and choose if they will pick-up the prescription personally or if it should be sent to a local pharmacy. The methodology also contains an attribute, which is used for storing the date of the last examination. Therefore, the system knows if the patient should ask for a prescription by wire or not. 

Finally, there is a reminder mechanism. Patients should set their primary contact information, which is used for the reminder service. Reminders can take the role of examination appointments, or prescription requests, or as part of the regular—preventive examination process. Furthermore, reminders should be set on the general system level in case of preventive examinations. This means that a national authority that deals with system management can manage preventive examination tasks. 

### 3.1. Methodology Implementation Case Study

The following text will describe using methodology for selected tasks, which are available to patients. The selected task will be described in the form of sequences, which illustrate the state of each component and how the component performs in tasks. 

**Figure 4:**
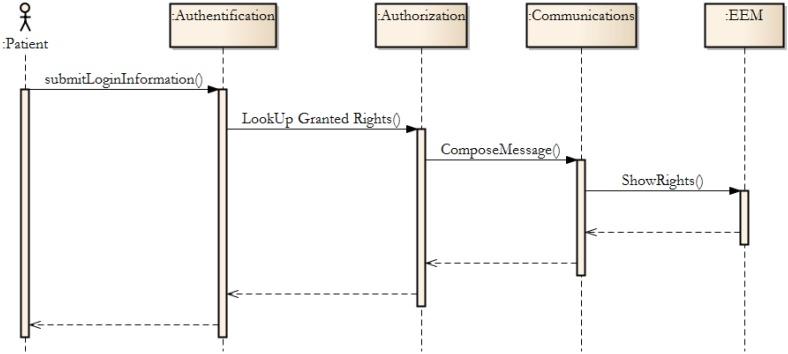
The authorization process in EEM.

In Figure 4, the authorisation process from the EEM is shown. Authorisation grants are managed by the EEM component, which is composed of the blocks mentioned in Figure 1. Synchronous messages are used. Therefore, the actor “Patient” receives a feedback response, if any error occurs. 

Patients can typically request a prescription. The Prescription Request Block (see Figure 1) is performed. The authorization process is used, and the following process starts. 

**Figure 5:**
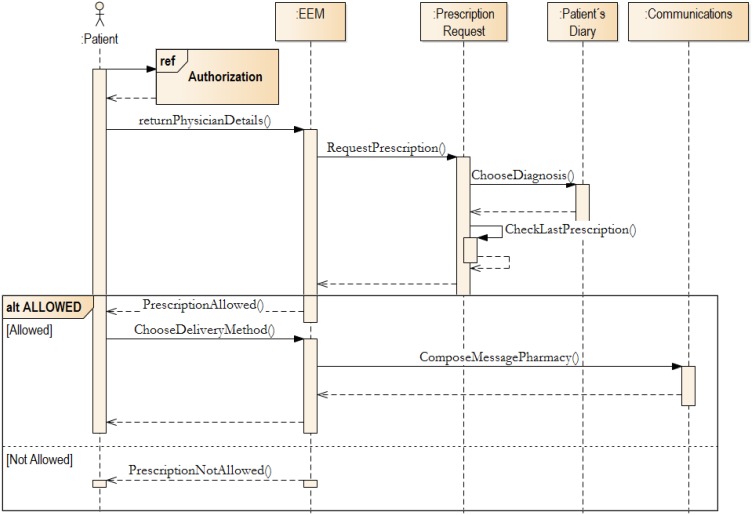
Prescription request implementation.

If Authorization is granted, the EEM will look-up, in the Prescription Request block - by using the Patient Diary function, the diagnosis. The patient chooses the diagnosis and then the limit of prescriptions will be checked. If the prescription limit has not been reached, then the patient’s request is submitted to the selected health-care provider queue. This activity is an intra-block activity (in EEM), and therefore cannot be seen in Figure 5. In the EEM, the key block is the Patient’s Diary. This block is key component, which requested by patients (see Section 2). Moreover, all other activities can be derived from the diary entry. 

In EEM the key block is the Patient Diary. This block is a key component, which was requested by patients (see Section 2—Methodology Prerequisites). Moreover, all other activities can be derived from the diary entry. A Patient adds textual information—e.g., topic, description. Supplementary files, like photographs or data files, can be added. When the entry is submitted, the system sends a message to the Communication Block, which dispatches a notification to the selected provider. The task is added to the health-care provider’s task queue and the provider informs patients that their entry is under investigation. 

### 3.2. Methodology Implementation Visualization

**Figure 6:**
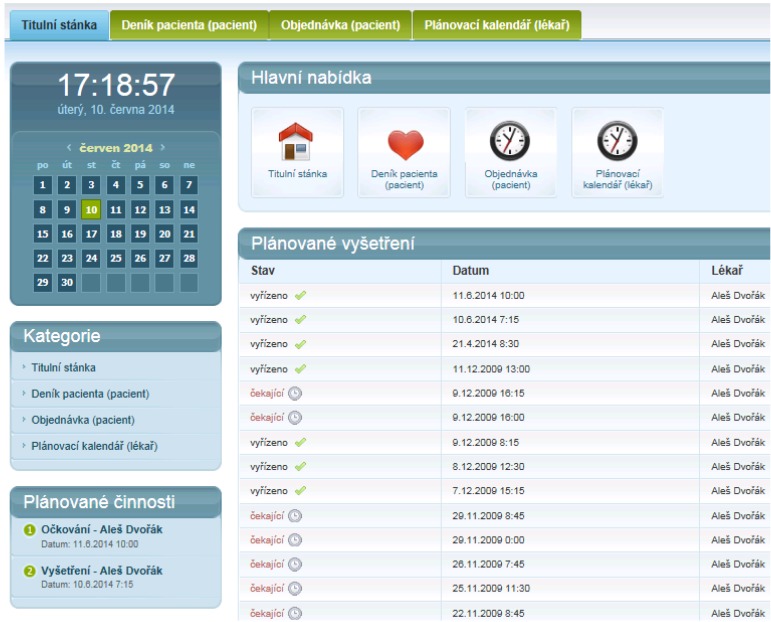
Patient dashboard.

The EEM methodology was used when the experimental application was designed. In the application there is prototype implementation of EEM. We propose a system which has a rich user interface. In the Figure 6, a Patients Dashboard is shown. The Patients Dashboard contains several functional blocks. Scheduled Examination, Delivered Messages and several other blocks can be seen in the Patients Dashboard. Examination request form is placed in the Patients Dashboard for fast access to the examination scheduling system. 

**Figure 7:**
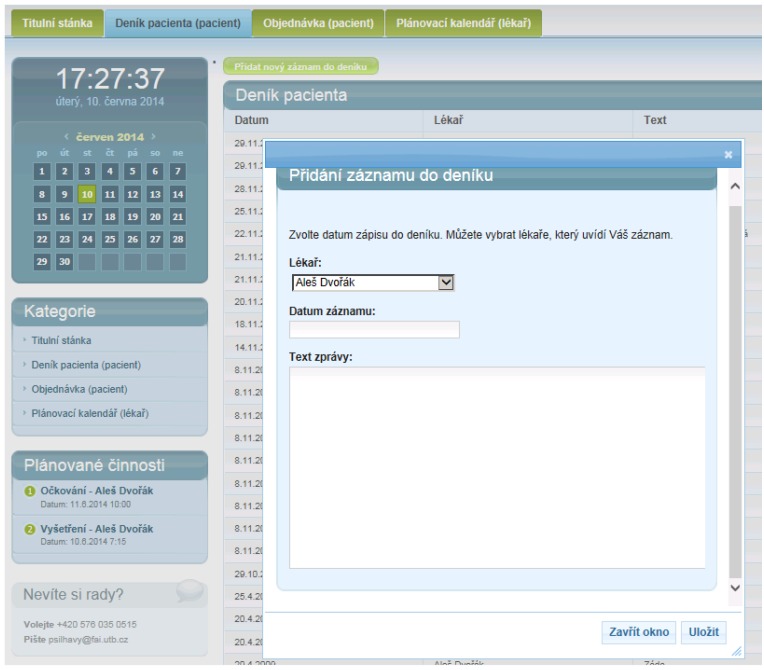
Patient’s diary entry list.

**Figure 8:**
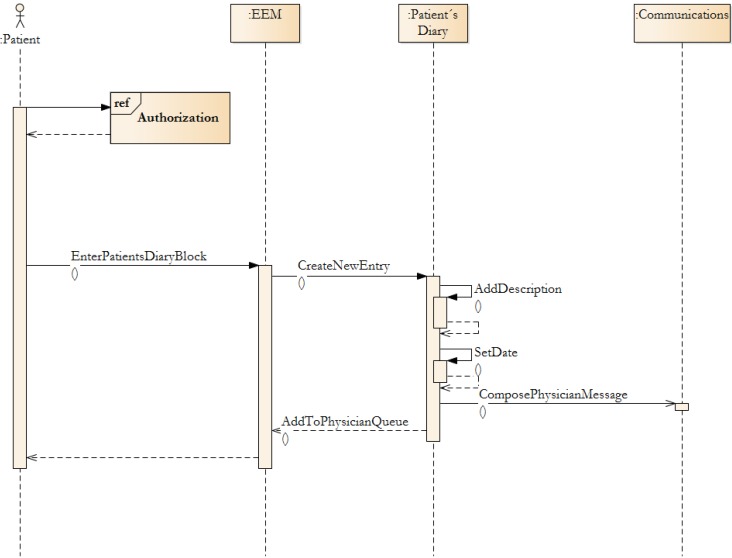
Patient diary entry submission process.

In the Figure 7, there can be seen a list of patient’s diary entries. These entries are submitted according the process, which is described in Figure 8. The Patient’s Diary is created in 1:N principles. More physicians can submit replies to patient’s queries. 

### 3.3. Prescription Request Evaluation

The study was conducted in the form of interviews in several physicians’ offices. The patients were asked to describe the travel time, waiting time and possible travel time to a pharmacy. Patients, which were available for comparison as user testers of proposed EEM were invited to the testing facility. Later in the Results section, all data are valid for those who attend the testing facility and taking a role as User Tester. Each tester was informed about the task by reading a written instruction only. This was because of the simulation of a real world environment. System Working Time was measured for each user tester. 

The System Working Time was measured when the user tester started the working process and measurement was stopped when the request was registered by the experimental system. Patients have requested simplification of the prescription process, as we mentioned early in the text. This process takes a significant role for chronically medicated patients. Therefore, we have chosen the Prescription Request, which is described in Figure 5, for evaluation. 

**Figure 9:**
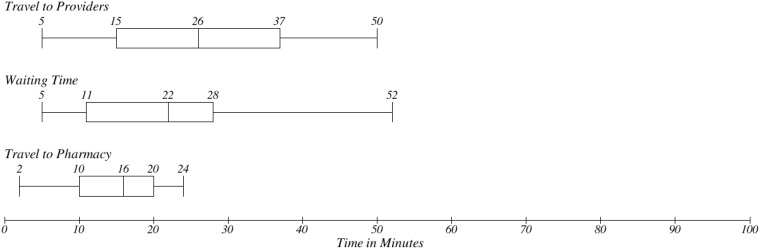
Patient’s time needs for basic steps in traditional concept.

**Figure 10:**
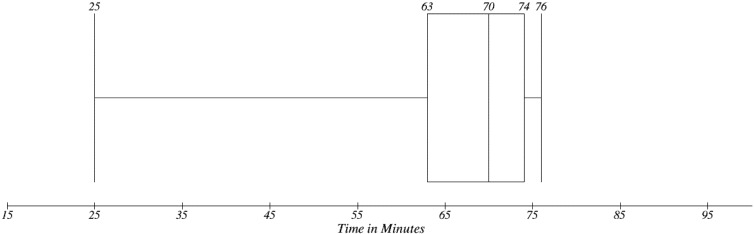
Overall time needs in traditional concept.

The traditional process of medication prescription in the scope of the Central European (see Section 2.1—Methodology Design) environment is based on the physical presence of the patients. There are three typical steps investigated in prescription process. Firstly, there is getting to the provider, secondly waiting time in waiting room and thirdly travelling to a pharmacy. Time needs in Figure 9 are based on our observation. In Figure 10, there is a distribution of overall time consumption for the whole prescription process. Ten samples were selected from the interviewed population. These patients were available for comparison as user testers of the proposed EEM. As can be seen the median value is the overall time needed to complete the process is 70 min. Travel to providers takes 26 min and waiting in the waiting room takes usually 22 min. 

**Figure 11:**
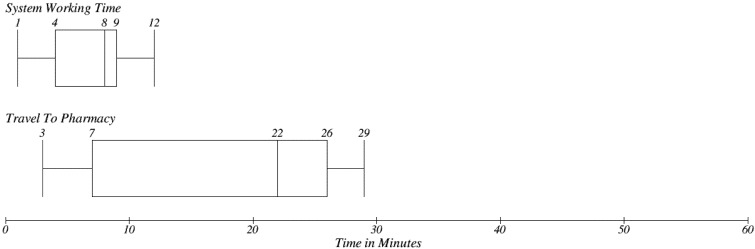
Patient’s time needs for basic steps in EEM.

The electronic prescription system is based on simplification of this process (see Figure 5). Patients should submit their request by using a software implementation, which will follow the EEM recommendations. There is a significant decrease in the time needed for the process. In Figure 11 one can be seen a time value for only two steps, which are performed on the patients’ site. The first one is System Working Time, which represents a Request Prescription use case (see Figure 2). A patient was asked to follow a use case, which was finished by submission of a request and the time needed to travel to the pharmacy were investigated. There is no need to visit a provider site and therefore there is no need to wait in the waiting room. The Median for System working time is 8 min, which depends on the patient’s level of computer familiarity. Travel to Pharmacy represents the time which is need to pick up a prescribed medicine. The time need for travel between the patient’s home and the pharmacy were investigated. 

**Figure 12:**
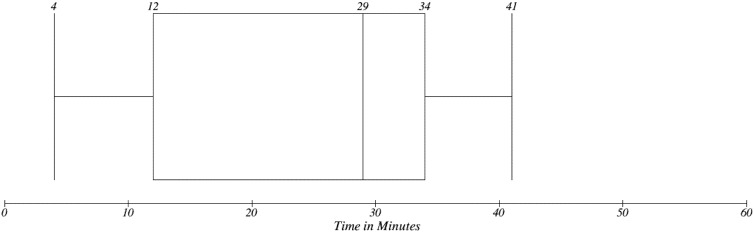
Overall time need in EEM.

Overall results can be seen in Figure 12. It can be seen that by adopting EEM the time consumption is reduced significantly. If we compare median values, it represents more than a 50% reduction. 

There are other factors, which have to be considering in the effectiveness evaluation of EEM. Many patients with disabilities or chronically sick ones may have serious transportation issues. Furthermore the time that patients stay in the waiting room, could be invested in some economic activity. More of the providers’ time could be used for examinations too. 

## 4. Conclusions

The main contribution of the proposed methodology can be found in its creating a set of recommendations to influence provider-patient systems design. The patient’s key tasks were described the article. The EEM resolves the complex set of tasks and aspects of patients-provider communication. The described methodology should be used as a guideline for the integration of heterogeneous systems, because of platform independency. Moreover, existing systems for sharing medical records can be integrated by using the proposed set of web services. 

The methodology presented herein represents a complex set of interconnected blocks which offer a synergistic effect. Both sides of the process can add or edit information and respond to data input. The methodology is characterized by the implementation of Effective Electronic Communication as the central layer. This layer is based on a simplified authentication and authorization method. Each individual subject—the patient or provider, has a unique identity. Therefore, it is possible to aggregate information about patients from many information systems or data stores. 

Effectiveness of evaluation was investigated in a user experiment. Using 10 samples we have tested the electronic prescription use case. The traditional prescription concept and method implemented in EEM were compared. EEM was found to be about 50 % more effective. Therefore EEM was found to be important for saving patients’ and providers’ time and other resources. Further research will be focused on creating bridges between legacy data formats, modern data formats (e.g., HL7) or the possibility of how aggregated data can improve diagnostic accuracy and how can economic benefits for healthcare can affected. 
